# Epidemiological dynamics of dengue in Peru: Temporal and spatial drivers between 2000 and 2022

**DOI:** 10.1371/journal.pone.0319708

**Published:** 2025-03-19

**Authors:** Katherine Susan Rufasto Goche, María Victoria Lizarbe Castro, Glenn Alberto Lozano Zanelly, Washington Melvin Lira Camargo, Elizabeth Yovana Ascayo Velasquez, Alexis G. Murillo Carrasco, Daysi Diaz-Obregón

**Affiliations:** 1 Faculty of Dentistry, Universidad Nacional Federico Villarreal, Lima, Peru; 2 Postgraduate School, Universidad Nacional Federico Villarreal, Lima, Peru; 3 Postgraduate School, Universidad Privada San Juan Bautista, Lima, Peru; 4 Faculty of Medicine, Universidad Nacional Federico Villarreal, Lima, Peru; 5 Immunology and Cancer Research Group-IMMUCA, OMICS, Lima, Peru; 6 ONG Innovation and Science for the Care and Support of Society–INNOVACARE, Lima, Peru; Instituto Nacional de Salud Publica, MEXICO

## Abstract

Dengue, a vector-borne disease driven by climate change, urbanization, and the adaptation of its vector, *Aedes aegypti*, poses a significant public health challenge. This study analyzed 23 years (2000–2022) of epidemiological data from Peru to examine the temporal and spatial drivers influencing dengue reported cases. Using secondary data from the Peruvian Ministry of Health, 501,027 cases were stratified by clinical severity, gender, geographic distribution, and temporal trends. The Amazonian and coastal regions, particularly Loreto, Ucayali, and Piura, bore the highest burden, collectively accounting for more than 60% of cases during epidemic years. Seasonal spikes in transmission consistently aligned with the rainy season, underscoring the dependence of *Aedes aegypti* proliferation on climatic conditions. The analysis revealed progressive geographic expansion of dengue into previously low-risk areas, including Andean highlands and peri-urban zones, driven by climatic shifts and unregulated urbanization. Gender-based comparisons indicated a higher overall case burden among females, though severe forms of dengue were unequally observed in males, particularly in rural areas. Clinical classifications highlighted that 78% of cases presented without warning signs, 18% with warning signs, and 4% as severe dengue. Socioeconomic factors, such as inadequate sanitation, urban slums, and water storage practices, contributed significantly to vector breeding in high-burden areas. Moreover, the role of extreme climatic events, including El Niño, exacerbated outbreak intensity and duration. The findings emphasize the urgent need for innovative approaches and provides actionable insights into regional dynamics and highlights critical areas for research, including predictive climate-disease modeling and the integration of molecular surveillance. These strategies are essential to mitigating the growing dengue burden and strengthening public health systems in Peru and similar endemic regions.

## Introduction

Dengue is a viral disease caused by the dengue virus (DENV), an arbovirus belonging to the *Flaviviridae* family, primarily transmitted by mosquitoes of the *Aedes* genus, especially *Aedes aegypti* and *Aedes albopictus*. This disease poses a critical public health challenge due to its rapid geographical spread, high prevalence, and diverse clinical manifestations, ranging from mild cases to severe forms that can be fatal without timely intervention [1]. According to the World Health Organization (WHO), more than half of the global population resides in areas at risk of dengue transmission, with an estimated 390 million infections annually, 96 million of which develop clinical symptoms [2].

The burden of dengue is particularly severe in tropical and subtropical regions, where climatic conditions—high temperatures and elevated humidity—favor mosquito breeding. However, over the past decades, dengue has expanded into previously non-endemic areas, driven by factors such as climate change, globalization, and increased human mobility. These dynamics have facilitated the proliferation of *Aedes aegypti* and *Aedes albopictus* in regions once free of the disease [3]. Additionally, rapid and unplanned urbanization, inadequate sanitation infrastructure, and the accumulation of stagnant water have intensified the risk of outbreaks, particularly in developing countries.

In Peru, dengue re-emerged as an endemic concern following the reintroduction of *Aedes aegypti* mosquitoes in 1984, after their eradication in 1956. Since then, recurrent outbreaks have been documented, primarily in Amazonian and coastal regions such as Loreto, Piura, and Tumbes. In 2023, the country faced one of its most severe epidemics, reporting over 200,000 cases and 200 deaths in the first half of the year, prompting a nationwide epidemiological alert [4]. These regions exhibit optimal conditions for dengue transmission due to climatic factors, such as intensified rainfall linked to El Niño, and socioeconomic factors, including limited basic infrastructure and insufficient access to sanitation services [5].

Controlling dengue poses significant challenges, including the lack of specific antiviral treatments, limitations in early diagnostic capacity, and growing resistance of mosquito vectors to conventional insecticides [6]. Furthermore, the restricted availability and efficacy of existing vaccines—requiring prior exposure to the virus to be safe and effective—have hindered preventive measures [7]. This scenario underscores the urgent need for innovative approaches, such as genetic technologies to modify vectors or multivalent vaccines that provide protection against all four virus serotypes [8].

This study aims to analyze the spatial and temporal distribution of dengue in Peru from 2000 to 2022, focusing on the regional and sociodemographic factors influencing its case count. By identifying key patterns and generating actionable insights, the research seeks to enhance the effectiveness of prevention and control strategies, particularly in vulnerable populations, while strengthening the capacity of the public health system to respond effectively to outbreaks.

## Methods

### Study design

This study utilized an observational, retrospective design based on secondary data analysis of the Peruvian Open Data Platform, specifically of the Dataset of dengue case notifications to the public health surveillance system in Peru. This dataset contains notifications of dengue cases to the public health surveillance system in Peru. It is managed and administered by the National Center for Epidemiology, Prevention, and Disease Control (CDC Peru). All analyses strictly adhered to these conditions and ensured the anonymization of any potentially identifiable data to protect individual privacy and maintain ethical research standards. The primary focus was on the temporal and spatial distribution of dengue cases reported across Peru from 2000 to 2022.

### Data source and collection

The data were extracted from an open-access epidemiological database provided by the Peruvian government (datosabiertos.gob.pe). The dataset includes 13 key variables, encompassing demographic, geographic, and clinical information for dengue cases confirmed by laboratory tests and disease severity based on clinical diagnosis.

To ensure data integrity, only records with complete demographic and epidemiological details (e.g., sex and suspected site of infection) were included. Variables such as epidemiological week, suspected site of infection, and clinical classification of dengue (with or without warning signs, severe dengue) were standardized for analysis.

### Variables

This dataset included variables for epidemiological, clinical, and molecular detection profiles.

Epidemiological Profile Variables included Epidemiological Week (Sequentially numbered weeks in a year when cases were reported), presumed Infection Site (Geographic location attributed to infection onset), and Sex (Male or female).

Clinical Variables included symptoms (defined based on patient-reported data) and Clinical Classification (classified as dengue without warning signs, with warning signs, or severe dengue).

Finally, the molecular detection profile was represented by laboratory results showing the presence of virus by rapid detection test outcomes.

### Data analysis

Basic descriptive statistics were applied to summarize demographic and clinical data. Frequencies and proportions were calculated for categorical variables, while means (±SD) were used for continuous variables with a normal distribution. Data normality was assessed using the Kolmogorov-Smirnov test. Depending on their distribution, quantitative data were analyzed using Student’s t-tests (parametric) or Mann-Whitney U test (non-parametric variables). Qualitative data was compared using the Chi-square test. Ordinal Logistic Regression was performed using a model to estimate the log-odds of an ordinal outcome (Dengue severity) as a function of categorical (region) and numerical predictors (year and epidemiological week). A geographic density map was created based on epidemiological records, using R software (version 4.3.2). The map visualized hotspots of dengue transmission across the Peruvian territory, highlighting areas with elevated number of cases in the frame 2000-2022.

### Ethical considerations

As this study used publicly accessible secondary data (from medical records stored in a public Peruvian repository), ethical approval was not required. Data stored in the Open Data Platform (https://www.datosabiertos.gob.pe/) is open access, fully anonymized, and available to any researcher. All datasets are publicly available and comply with transparency and free-use principles outlined by the platform’s terms of use. All analyses adhered to ethical guidelines for research involving human data, ensuring data processing with confidentiality.

## Results

### Clinical characteristics and demographics

The clinical spectrum of dengue cases showed that 78% were classified as dengue without warning signs, 18% with warning signs, and 4% as severe dengue. Gender analysis indicated that females represented 53% of cases, with higher proportions in urban and peri-urban settings, whereas males dominated in rural and occupational exposure contexts. The classification of rurality across Peruvian regions was based on data from the 2017 National Census conducted in Peru [9]. Given these demographic patterns, we next examined the temporal trends in dengue cases to assess whether incidence has changed over time.

### Temporal trends in dengue reported cases (2000–2022)

**Fig 1:**
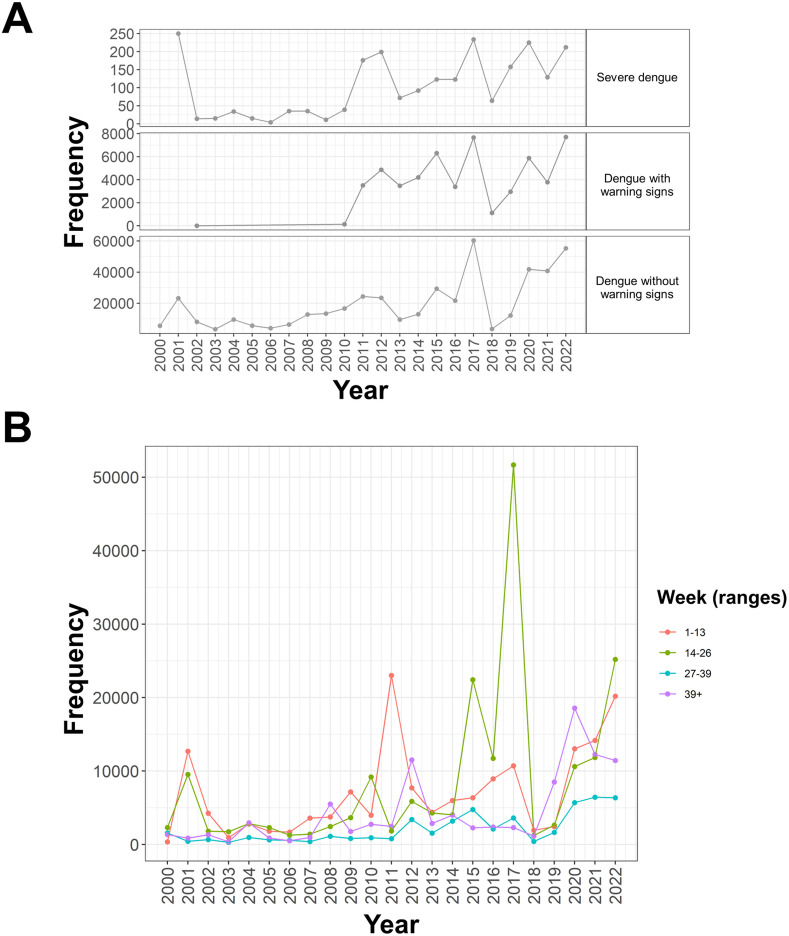
Year-by-year trend of dengue cases in Peru (2000-2022). Time trends highlighting seasonal spikes classified by (A) disease severity or (B) epidemiological weeks. Data from the Peruvian Open Data Platform, Dataset of dengue case notifications to the public health surveillance system in Peru.

Dengue cases in Peru exhibited a progressive increase over the 23 years studied, characterized by marked periodic peaks during epidemic years, such as 2017 and 2023 (Fig 1A). Seasonal spikes were consistently observed between epidemiological weeks 14 and 26 (Fig 1B). To better understand how these trends vary across different regions of Peru, we conducted a spatial analysis of dengue case distribution.

### Regional and spatial patterns

**Fig 2:**
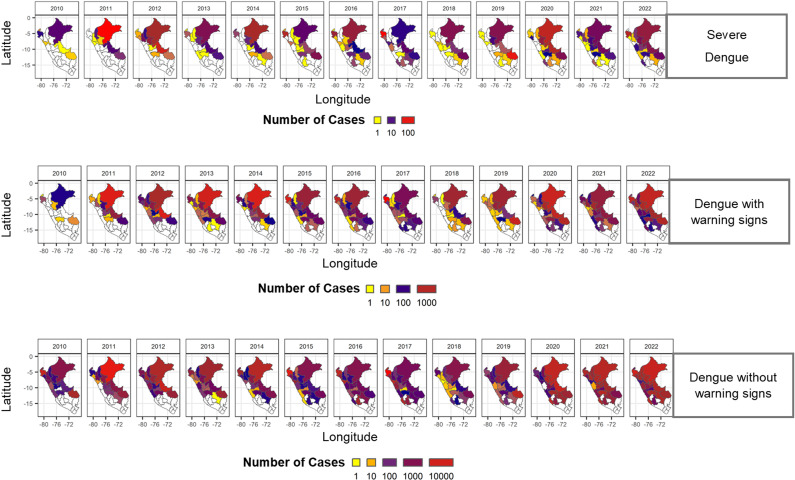
Spatial distribution of dengue cases in Peru during 2000–2022. Heatmap showing clusters with higher number of dengue cases in Peruvian regions. Cases are stratified according to the disease severity (Severe dengue, Dengue with warning signs, and Dengue without warning signs). Data from the Peruvian Open Data Platform, Dataset of dengue case notifications to the public health surveillance system in Peru.

Spatial analysis revealed significant disparities in dengue cases across Peru. Departments in the Amazon basin, including Loreto and Ucayali, consistently reported high number of cases (Fig 2 and S1 Table), driven by year-round favorable environmental conditions such as high humidity and temperatures. These regions accounted for more than 40% of the national burden during epidemic years. Coastal regions, such as Piura, Lambayeque, and Ica, experienced periodic outbreaks, exacerbated by El Niño events that increased rainfall and flooding. Given the observed spatial disparities, we further analyzed the weekly distribution of cases to determine whether specific time periods within the year showed consistent surges in infections.

### Weekly distribution and epidemic dynamics

Epidemiological week analysis demonstrated a pronounced clustering of cases during the rainy season, with the highest number of reported cases between weeks 16 and 24 (Fig 1B). This pattern aligns with the environmental conditions that facilitate mosquito breeding and virus transmission.

To statistically validate these epidemiological patterns, we compared the distribution of dengue cases across different demographic and geographic groups.

### Statistical inferences

Statistical comparisons of dengue cases across different departments, genders, and clinical forms can be observed at Table 1. Significant differences (p < 0.001) are observed in the distribution of cases between males and females, with women generally showing a higher proportion of severe cases. This trend is particularly notable in departments such as Loreto and Piura (Table 1 and Fig 2). In addition, Table 2 shows the results of a logistic regression model evaluating the impact of region, year, and epidemiological week in the reporting of dengue cases. Compared to Lima, several regions exhibit significantly higher odds, notably Ucayali (OR =  3.05, 95% CI: 2.68–3.47), Madre de Dios (OR =  3.02, 95% CI: 2.65–3.43), and Loreto (OR =  2.92, 95% CI: 2.57-3.31). Conversely, Lambayeque (OR =  0.29, 95% CI: 0.25–0.35) and Cusco (OR =  0.34, 95% CI: 0.29–0.41) show markedly lower odds. The odds increase significantly over time, with 2011–2016 (OR =  31.0, 95% CI: 27.8–34.6) and 2017–2022 (OR =  24.4, 95% CI: 21.8–27.2) showing the highest values. Relative to epidemiological weeks 1–13, weeks 27–39 exhibit a slight increase (OR =  1.07, 95% CI: 1.03–1.10), whereas week 39 + shows a marginal decrease (OR =  0.97, 95% CI: 0.94–1.00).

**Table 1:** Statistical comparison of epidemiological characteristics with dengue severity in Peru (2000–2022).

Variable	N	Dengue without warning signs	Dengue with warning signs	Severe dengue	p-value^*2*^
N = 443,791^*1*^	N = 54,977^*1*^	N = 2,259^*1*^
**Region**	501,027				<0.001
AMAZONAS		11,476/ 443791 (2.6%)	502/ 54977 (0.9%)	39/ 2259 (1.7%)	
ANCASH		6,899/ 443791 (1.6%)	296/ 54977 (0.5%)	19/ 2259 (0.8%)	
AYACUCHO		8,600/ 443791 (1.9%)	269/ 54977 (0.5%)	20/ 2259 (0.9%)	
CAJAMARCA		17,503/ 443791 (3.9%)	1,869/ 54977 (3.4%)	37/ 2259 (1.6%)	
CUSCO		10,184/ 443791 (2.3%)	270/ 54977 (0.5%)	22/ 2259 (1.0%)	
HUANUCO		8,000/ 443791 (1.8%)	1,012/ 54977 (1.8%)	34/ 2259 (1.5%)	
ICA		20,709/ 443791 (4.7%)	583/ 54977 (1.1%)	32/ 2259 (1.4%)	
JUNIN		17,102/ 443791 (3.9%)	2,032/ 54977 (3.7%)	65/ 2259 (2.9%)	
LA LIBERTAD		23,392/ 443791 (5.3%)	948/ 54977 (1.7%)	37/ 2259 (1.6%)	
LAMBAYEQUE		15,368/ 443791 (3.5%)	217/ 54977 (0.4%)	33/ 2259 (1.5%)	
LIMA		3,988/ 443791 (0.9%)	263/ 54977 (0.5%)	4/ 2259 (0.2%)	
LORETO		77,828/ 443791 (18%)	14,137/ 54977 (26%)	531/ 2259 (24%)	
MADRE DE DIOS		25,098/ 443791 (5.7%)	5,208/ 54977 (9.5%)	256/ 2259 (11%)	
PASCO		1,742/ 443791 (0.4%)	205/ 54977 (0.4%)	16/ 2259 (0.7%)	
PIURA		113,287/ 443791 (26%)	12,604/ 54977 (23%)	511/ 2259 (23%)	
SAN MARTIN		22,446/ 443791 (5.1%)	4,162/ 54977 (7.6%)	151/ 2259 (6.7%)	
TUMBES		24,197/ 443791 (5.5%)	2,995/ 54977 (5.4%)	122/ 2259 (5.4%)	
UCAYALI		35,972/ 443791 (8.1%)	7,405/ 54977 (13%)	330/ 2259 (15%)	
**Year (grouped)**	501,027				<0.001
2000-2005		55,376/ 443791 (12%)	1/ 54977 (<0.1%)	328/ 2259 (15%)	
2006-2010		53,178/ 443791 (12%)	134/ 54977 (0.2%)	124/ 2259 (5.5%)	
2011-2016		121,343/ 443791 (27%)	25,741/ 54977 (47%)	785/ 2259 (35%)	
2017-2022		213,894/ 443791 (48%)	29,101/ 54977 (53%)	1,022/ 2259 (45%)	
**Epidemiological Week (ranges)**	501,027				<0.001
1-13		142,864/ 443791 (32%)	18,152/ 54977 (33%)	844/ 2259 (37%)	
14-26		173,146/ 443791 (39%)	18,055/ 54977 (33%)	696/ 2259 (31%)	
27-39		41,940/ 443791 (9.5%)	6,136/ 54977 (11%)	212/ 2259 (9.4%)	
39+		85,841/ 443791 (19%)	12,634/ 54977 (23%)	507/ 2259 (22%)	
**Sex**	501,027				<0.001
Female		235,049/ 443791 (53%)	30,273/ 54977 (55%)	1,236/ 2259 (55%)	
Male		208,742/ 443791 (47%)	24,704/ 54977 (45%)	1,023/ 2259 (45%)	

^1^n/ N (%).

^2^Pearson’s Chi-squared test.

**Table 2:** Adjusted odds ratios for variables related to dengue cases in this study.

Variable	OR	95% CI	p-value
**Region**			
LIMA	—	—	
UCAYALI	3.05	2.68, 3.47	<0.001
MADRE DE DIOS	3.02	2.65, 3.43	<0.001
LORETO	2.92	2.57, 3.31	<0.001
SAN MARTIN	2.59	2.28, 2.95	<0.001
HUANUCO	1.93	1.68, 2.23	<0.001
TUMBES	1.82	1.60, 2.08	<0.001
CAJAMARCA	1.81	1.58, 2.07	<0.001
PIURA	1.76	1.55, 2.00	<0.001
PASCO	1.61	1.33, 1.94	<0.001
JUNIN	1.6	1.40, 1.82	<0.001
LA LIBERTAD	0.79	0.69, 0.91	0.001
AMAZONAS	0.78	0.67, 0.91	0.001
ANCASH	0.61	0.52, 0.73	<0.001
AYACUCHO	0.39	0.33, 0.46	<0.001
ICA	0.36	0.31, 0.42	<0.001
CUSCO	0.34	0.29, 0.41	<0.001
LAMBAYEQUE	0.29	0.25, 0.35	<0.001
**Year**			
2000-2005	—	—	
2006-2010	0.69	0.58, 0.81	<0.001
2011-2016	31	27.8, 34.6	<0.001
2017-2022	24.4	21.8, 27.2	<0.001
**Epidemiological week**			
1-13	—	—	
14-26	1.03	1.00, 1.05	0.037
27-39	1.07	1.03, 1.10	<0.001
39+	0.97	0.94, 1.00	0.02

CI =  Confidence Interval, OR =  Odds Ratio.

## Discussion

This study offers a comprehensive analysis of dengue epidemiology in Peru over a 23-year period, providing valuable insights into the temporal, geographic, and demographic dynamics of the disease. The findings highlight the intricate interplay of environmental, social, and biological factors driving dengue transmission in the region.

Dengue cases have steadily increased, with major outbreaks recorded in 2011–2012, 2017, and 2020. These peaks coincided with extreme climatic phenomena, such as El Niño, which exacerbated rainfall and temperature fluctuations, creating favorable conditions for vector proliferation [10,11], particularly in the period 2010-2011 [12] where our study found a notable rise in dengue cases with warning signs. Climatic and environmental factors played a major role in this outbreak, as consequence of the climate change events over the past decades [13]. Rising global temperatures also accelerated the biological cycle of mosquitoes and enhanced the conditions for virus transmission [14].

This reinforces the established link between climate variability and the dynamics of vector-borne diseases, emphasizing the urgent need to adapt public health responses to these changing conditions. Moreover, the emergence of year-round transmission in historically seasonal regions reflects shifts in climatic patterns, narrowing the temporal windows for effective vector control. High-burden regions, such as Loreto and Piura, experienced significant strain on healthcare systems during epidemic peaks, exposing the need for resilient and adaptive health infrastructure. Additionally, uncontrolled urban expansion and poor infrastructure for managing wastewater and solid waste created environments that further enabled the proliferation of the mosquito vector. Social and demographic factors also contributed significantly to the spread of dengue. Moreover, increased travel and migration promoted the introduction and spread of new dengue serotypes, leading to secondary infections and severe cases [13].

The dengue virus (DENV) belongs to the Flaviviridae family and includes four serotypes that are relevant to human infections: DENV-1, DENV-2, DENV-3, and DENV-4 [15]. In Peru, the emergence of a new lineage of the American/Asian genotype DENV-2 at the end of 2010 led to numerous outbreaks and an epidemic, significantly increasing the demand for healthcare services [16,17]. In 2011, Peru recorded 22,087 cases of dengue without warning signs (88.5%), 2,720 cases with warning signs (10.9%), 158 cases of severe dengue (0.6%), and 29 deaths [16].

The severe form of dengue is closely associated with high morbidity and mortality. Factors such as extreme age, previous dengue infections, and the serotype of the virus have been strongly linked to the development of severe cases [18]. Furthermore, while the increase in reported cases can partly be attributed to a genuine rise in infections, it was also associated with improvements in detection and reporting systems in the recent years. The implementation of early warning systems based on climate forecasts is crucial for anticipating outbreaks and initiating preventive interventions, thereby strengthening the resilience of health systems in the face of climate change [19].

The seasonality of dengue in Peru, with a peak between April and June, reflects the impact of the rainy season on vector proliferation. Globally, dengue exhibits pronounced seasonality in tropical and subtropical regions, influenced by climatic factors and human mobility. During warm and humid months, mosquito populations increase, intensifying virus transmission [20].

The spatial analysis revealed substantial geographic disparities in dengue case reporting. Regions in the Amazon basin, including Loreto, San Martín, and Ucayali, and coastal areas such as Piura, Lambayeque, and Ica bore a disproportionate burden of cases. Coastal regions experienced periodic outbreaks exacerbated by flooding and inadequate waste management, while urban slums emerged as hotspots for mosquito breeding due to stagnant water in unregulated reservoirs [21]. The rise of cases in peri-urban Lima, a historically non-endemic zone, underscores the role of poor water storage practices and inadequate infrastructure in facilitating vector habitat expansion. These findings illustrate the complex interplay of ecological, climatic, and socioeconomic factors that drive dengue transmission and highlight the need for tailored, region-specific prevention strategies.

The immune response to dengue may vary by sex, with evidence suggesting that women experience a more intense inflammatory response, making them more susceptible to complications [22]. While it has been noted that women tend to have a higher proportion of severe cases, men are slightly more affected by severe dengue overall.

This apparent discrepancy may be explained by several factors. First, occupational exposure is a significant contributor. In many regions, men are more likely to engage in outdoor or labor-intensive work environments, increasing their exposure to mosquito bites. This heightened risk due to occupational settings explains the higher number of dengue infections in males in specific demographics. Second, differences in access to healthcare could play a role. Cultural and gender norms may result in men seeking medical care later than women, potentially leading to a higher likelihood of complications. Lastly, biological factors, such as sex-specific immunological differences, may influence how dengue progresses toward severe forms in men versus women.

Consistently with these hypotheses, Ahmad et al. (2018) analyzed data from a dengue outbreak in Punjab, Pakistan, using records of 21,000 patients, primarily from Lahore, the epicenter of the outbreak [23]. The study revealed that men aged 30 years were most severely affected due to their involvement in business and office work, which exposed them to the virus. Geographically, men aged 90 years experienced the widest impact, covering 68% of the study area, though their infection intensity was minimal. Women aged 30 years were the most affected among females, covering 47% of the geographical area, which was 21% less than that of men. Men accounted for 14,363 cases, compared to 6,725 cases among women, underscoring a higher prevalence in males.

Similarly, Yue et al. (2019) investigated the epidemiological dynamics of dengue from 2014 to 2018, analyzing imported and indigenous cases [24]. The study found that men consistently exhibited higher rates of dengue infection, with an annual male-to-female ratio of approximately 60:40. Occupational roles such as farmers and businessmen showed the highest rates of imported cases, suggesting that exposure through work environments contributed significantly to these gender disparities. Therefore, while both men and women are affected by dengue, men tend to have higher overall prevalence rates due to increased exposure through occupational activities and potential delays in seeking medical attention. Naranjo-Gómez et al. (2020) reported that women are more prone to severe inflammatory responses, which may lead to health complications [22].

In addition, there is important to consider the phenomenon of antibody-dependent enhancement [25], where prior exposure to dengue virus serotypes increases the severity of subsequent infections [26]. The prominence of severe cases in Amazonian regions points to gaps in timely access to healthcare and suggests the influence of genetic or virulence factors, particularly associated with DENV-2, a serotype linked to severe outcomes in other Latin American contexts [27]. These demographic vulnerabilities highlight the importance of targeted interventions and improved access to healthcare in high-burden areas.

The findings also underscore the limitations of existing dengue control strategies, including insecticide-based fumigation and public awareness campaigns. These approaches face challenges such as insecticide resistance, logistical constraints, and limited efficacy in reducing the number of cases, particularly in socioeconomically disadvantaged regions [9]. Addressing these gaps requires innovative and integrated approaches. Biotechnological advances, such as Wolbachia-infected mosquitoes [28] and genetically modified *Aedes aegypti* [29], have shown promise in global pilot studies and could be adapted to Peru’s context. Expanding vaccine access, including Dengvaxia [26], Butantan-DV [30] and next-generation tetravalent vaccines, offers a complementary layer of protection, particularly for populations with prior dengue exposure.

While this study provides robust insights into the epidemiological patterns of dengue in Peru, some limitations must be acknowledged. The reliance on passive surveillance data may have led to underreporting, particularly in rural regions with limited healthcare access. The absence of serotype-specific and regional population at-risk data restricts the ability to evaluate the influence of viral genetics on disease dynamics and calculating incidence rates. Additionally, the lack of socioeconomic, climatic, and behavioral data of the suspected sites of infection limits the exploration of broader risk factors influencing dengue transmission. Future research should prioritize the integration of climate-disease modeling to predict outbreaks, genomic surveillance to monitor serotype evolution, and longitudinal evaluation of novel interventions such as Wolbachia-based strategies and advanced vaccines. These efforts will be crucial to mitigating the growing burden of dengue and ensuring equitable healthcare delivery in Peru and other endemic regions.

## Conclusion

The dengue situation in Peru is continuously evolving, driven by a complex interplay of climatic, socioeconomic, and biological factors. This study highlights the urgent need for more comprehensive and innovative public health strategies, incorporating advanced vector control technologies, enhanced surveillance systems, and equitable access to healthcare.

Strengthening these pillars is not only crucial to reducing the burden of dengue in Peru but also to serving as a model for other endemic regions facing similar challenges. An effective response will depend on the ability to integrate interdisciplinary approaches, fostering the resilience of health systems, and protecting the most vulnerable populations from this ever-expanding disease.

## Supporting information

S1 Table Department-wise breakdown of dengue cases by year, clinical severity, and reported cases in Peru 2000–2022. (DOCX)
