# Supplementary material for: Epidemiological dynamics of dengue in Peru: Temporal and spatial drivers between 2000 and 2022
Source: PLoS One. 2025 Mar 19;20(3):e0319708. doi: 10.1371/journal.pone.0319708 (PMC11922284; doi:10.1371/journal.pone.0319708)
Supplement: S1 Table — (DOCX) [file pone.0319708.s001.docx]

**Table S1.** Department-wise breakdown of dengue cases by year, clinical severity, and incidence values in Peru 2000 - 2022.

| **Region** | **Disease Severity** | **2000** | **2001** | **2002** | **2003** | **2004** | **2005** | **2006** | **2007** | **2008** | **2009** | **2010** | **2011** | **2012** | **2013** | **2014** | **2015** | **2016** | **2017** | **2018** | **2019** | **2020** | **2021** | **2022** |
| --- | --- | --- | --- | --- | --- | --- | --- | --- | --- | --- | --- | --- | --- | --- | --- | --- | --- | --- | --- | --- | --- | --- | --- | --- |
| AMAZONAS | DENGUE WITHOUT WARNING SIGNS | 341 | 692 | 30 | 143 | 312 | 409 | 35 | 320 | 2 | 158 | 273 | 3 | 65 | 7 | 16 | 2 | 8 | 2 | 1 | 6 | 71 | 115 | 206 |
| AMAZONAS | DENGUE WITH WARNING SIGNS | *NA* | *NA* | *NA* | *NA* | *NA* | *NA* | *NA* | *NA* | 646 | *NA* | *NA* | 10 | 11 | 1 | 191 | 1 | 82 | 91 | 108 | 158 | 3 | 3 | 8 |
| AMAZONAS | SEVERE DENGUE | *NA* | *NA* | *NA* | *NA* | *NA* | *NA* | *NA* | *NA* | *NA* | *NA* | *NA* | 292 | 511 | 239 | *NA* | 34 | *NA* | *NA* | *NA* | *NA* | 799 | 2251 | 3361 |
| ANCASH | DENGUE WITH WARNING SIGNS | *NA* | 4 | 824 | 1 | 8 | 4 | 1 | 8 | 77 | 224 | 50 | *NA* | 19 | 20 | *NA* | 24 | 38 | 25 | 1 | 3 | *NA* | 14 | 153 |
| ANCASH | SEVERE DENGUE | *NA* | *NA* | *NA* | *NA* | *NA* | *NA* | *NA* | *NA* | *NA* | *NA* | *NA* | *NA* | 1049 | 434 | *NA* | 94 | 1 | 5 | 5 | 17 | *NA* | 1 | 11 |
| ANCASH | DENGUE WITHOUT WARNING SIGNS | *NA* | *NA* | *NA* | *NA* | *NA* | *NA* | *NA* | *NA* | *NA* | *NA* | *NA* | *NA* | *NA* | *NA* | *NA* | *NA* | 415 | 1690 | *NA* | *NA* | *NA* | 13 | 1981 |
| AYACUCHO | DENGUE WITH WARNING SIGNS | *NA* | *NA* | *NA* | *NA* | *NA* | *NA* | *NA* | *NA* | *NA* | *NA* | *NA* | *NA* | *NA* | 1 | *NA* | 21 | 18 | 70 | 11 | 1 | 38 | 36 | 74 |
| AYACUCHO | SEVERE DENGUE | *NA* | *NA* | *NA* | *NA* | *NA* | *NA* | *NA* | *NA* | *NA* | *NA* | *NA* | *NA* | *NA* | *NA* | *NA* | 1 | 7 | 6 | 191 | 94 | 4 | 1 | 708 |
| AYACUCHO | DENGUE WITHOUT WARNING SIGNS | *NA* | *NA* | *NA* | *NA* | *NA* | *NA* | *NA* | *NA* | *NA* | *NA* | *NA* | *NA* | *NA* | *NA* | *NA* | 246 | 2613 | 1581 | *NA* | *NA* | 1541 | 1626 | *NA* |
| CAJAMARCA | DENGUE WITHOUT WARNING SIGNS | 18 | 1100 | 1176 | 114 | 383 | 1 | 123 | 125 | 1 | 477 | 784 | 27 | 332 | 40 | 87 | 70 | 52 | 3 | 6 | 17 | 41 | 496 | 704 |
| CAJAMARCA | DENGUE WITH WARNING SIGNS | *NA* | *NA* | *NA* | *NA* | *NA* | 1126 | *NA* | *NA* | 463 | *NA* | *NA* | 1 | 23 | 1 | 208 | 148 | 229 | 417 | *NA* | 381 | 225 | 8 | 2 |
| CAJAMARCA | SEVERE DENGUE | *NA* | *NA* | *NA* | *NA* | *NA* | *NA* | *NA* | *NA* | *NA* | *NA* | *NA* | 660 | 2853 | 44 | *NA* | *NA* | *NA* | *NA* | *NA* | *NA* | *NA* | 3511 | 2932 |
| CUSCO | DENGUE WITH WARNING SIGNS | *NA* | *NA* | 2 | *NA* | *NA* | 2 | *NA* | *NA* | *NA* | *NA* | *NA* | 57 | *NA* | 1 | 3 | 21 | 49 | 57 | 5 | 4 | 60 | 16 | 54 |
| CUSCO | SEVERE DENGUE | *NA* | *NA* | *NA* | *NA* | *NA* | *NA* | *NA* | *NA* | *NA* | *NA* | *NA* | *NA* | *NA* | 1 | 1 | 227 | 2 | 8 | 2 | 2 | 3 | 1 | 3 |
| CUSCO | DENGUE WITHOUT WARNING SIGNS | *NA* | *NA* | *NA* | *NA* | *NA* | *NA* | *NA* | *NA* | *NA* | *NA* | *NA* | *NA* | *NA* | *NA* | 223 | *NA* | 1049 | 472 | 72 | 48 | 2436 | 1965 | 3630 |
| HUANUCO | DENGUE WITHOUT WARNING SIGNS | 29 | 159 | 1 | 107 | 356 | 143 | 128 | 28 | 1 | 1 | 214 | 38 | 77 | 15 | 35 | 85 | 177 | 26 | 5 | 5 | 97 | 233 | 219 |
| HUANUCO | DENGUE WITH WARNING SIGNS | *NA* | *NA* | 131 | *NA* | *NA* | *NA* | *NA* | *NA* | 109 | 260 | *NA* | 98 | 1 | 52 | 1 | 1 | 6 | 66 | 1 | 30 | 2 | 11 | 8 |
| HUANUCO | SEVERE DENGUE | *NA* | *NA* | *NA* | *NA* | *NA* | *NA* | *NA* | *NA* | *NA* | *NA* | *NA* | *NA* | 258 | *NA* | 93 | 221 | 545 | *NA* | 19 | *NA* | 1034 | 2567 | 1353 |
| ICA | DENGUE WITH WARNING SIGNS | *NA* | *NA* | *NA* | *NA* | *NA* | *NA* | *NA* | *NA* | *NA* | *NA* | *NA* | *NA* | *NA* | *NA* | *NA* | 3 | 3 | 98 | 4 | 4 | 144 | 112 | 218 |
| ICA | SEVERE DENGUE | *NA* | *NA* | *NA* | *NA* | *NA* | *NA* | *NA* | *NA* | *NA* | *NA* | *NA* | *NA* | *NA* | *NA* | *NA* | *NA* | 320 | 8 | 123 | 47 | 8 | 6 | 10 |
| ICA | DENGUE WITHOUT WARNING SIGNS | *NA* | *NA* | *NA* | *NA* | *NA* | *NA* | *NA* | *NA* | *NA* | *NA* | *NA* | *NA* | *NA* | *NA* | *NA* | *NA* | *NA* | 4278 | *NA* | *NA* | 6992 | 4090 | 4856 |
| JUNIN | DENGUE WITHOUT WARNING SIGNS | 7 | 48 | 207 | 116 | 192 | 114 | 189 | 1 | 8 | 245 | 3 | 6 | 41 | 86 | 40 | 223 | 243 | 26 | 9 | 80 | 431 | 400 | 444 |
| JUNIN | DENGUE WITH WARNING SIGNS | *NA* | *NA* | *NA* | *NA* | *NA* | *NA* | *NA* | 377 | *NA* | *NA* | 137 | 81 | 3 | 1 | 3 | 8 | 9 | 1 | 1 | 3 | 12 | 12 | 11 |
| JUNIN | SEVERE DENGUE | *NA* | *NA* | *NA* | *NA* | *NA* | *NA* | *NA* | *NA* | *NA* | *NA* | *NA* | *NA* | 692 | 694 | 465 | 543 | 679 | 193 | 41 | 399 | 3295 | 4678 | 3702 |
| LA LIBERTAD | DENGUE WITHOUT WARNING SIGNS | 1496 | 1 | 3 | *NA* | 263 | 259 | 10 | 11 | 267 | 134 | 2 | 1 | 7 | 7 | 2 | 148 | 357 | 383 | 2 | 15 | 8 | 3 | 18 |
| LA LIBERTAD | DENGUE WITH WARNING SIGNS | *NA* | 5717 | *NA* | *NA* | *NA* | *NA* | *NA* | 1471 | *NA* | *NA* | 726 | 16 | 97 | 16 | 61 | 5 | 3 | 10 | 1 | 1 | 382 | 1 | 144 |
| LA LIBERTAD | SEVERE DENGUE | *NA* | *NA* | *NA* | *NA* | *NA* | *NA* | *NA* | *NA* | *NA* | *NA* | *NA* | *NA* | *NA* | *NA* | *NA* | 1920 | 4290 | 5511 | *NA* | 350 | *NA* | 258 | *NA* |
| LAMBAYEQUE | DENGUE WITH WARNING SIGNS | *NA* | 813 | 45 | 79 | 1868 | 5 | 77 | 656 | 718 | 679 | 291 | 10 | 45 | 1 | 147 | 19 | 17 | 44 | 4 | 22 | 3 | 26 | 40 |
| LAMBAYEQUE | SEVERE DENGUE | *NA* | *NA* | *NA* | *NA* | *NA* | 799 | *NA* | *NA* | *NA* | *NA* | *NA* | *NA* | 4 | 24 | *NA* | 1084 | 2 | 6 | *NA* | 1 | 13 | 1 | 1 |
| LAMBAYEQUE | DENGUE WITHOUT WARNING SIGNS | *NA* | *NA* | *NA* | *NA* | *NA* | *NA* | *NA* | *NA* | *NA* | *NA* | *NA* | *NA* | 442 | *NA* | *NA* | *NA* | 1643 | 1529 | *NA* | 747 | 552 | 816 | 2345 |
| LIMA | DENGUE WITH WARNING SIGNS | *NA* | 2 | *NA* | *NA* | *NA* | 443 | 10 | 91 | *NA* | 235 | 90 | *NA* | 32 | 19 | 4 | 2 | 2 | 17 | 11 | 5 | 23 | 88 | 75 |
| LIMA | SEVERE DENGUE | *NA* | *NA* | *NA* | *NA* | *NA* | *NA* | *NA* | *NA* | *NA* | *NA* | *NA* | *NA* | 282 | 1 | *NA* | 7 | 56 | 345 | *NA* | 41 | 1 | 1102 | 2 |
| LIMA | DENGUE WITHOUT WARNING SIGNS | *NA* | *NA* | *NA* | *NA* | *NA* | *NA* | *NA* | *NA* | *NA* | *NA* | *NA* | *NA* | *NA* | 82 | *NA* | *NA* | *NA* | *NA* | *NA* | *NA* | 326 | *NA* | 861 |
| LORETO | DENGUE WITHOUT WARNING SIGNS | 518 | 1 | 1 | 784 | 5 | 4 | 3 | 18 | 7 | 1 | 85 | 2713 | 755 | 2032 | 2451 | 519 | 563 | 264 | 607 | 592 | 1688 | 468 | 1399 |
| LORETO | DENGUE WITH WARNING SIGNS | *NA* | 509 | 2499 | *NA* | 2575 | 1768 | 1992 | 1702 | 7225 | 3781 | 17 | 138 | 41 | 25 | 42 | 18 | 33 | 13 | 26 | 26 | 59 | 22 | 32 |
| LORETO | SEVERE DENGUE | *NA* | *NA* | *NA* | *NA* | *NA* | *NA* | *NA* | *NA* | *NA* | *NA* | 1220 | 18394 | 3586 | 2422 | 4556 | 1093 | 1090 | 812 | 1200 | 1929 | 6053 | 4625 | 7495 |
| MADRE DE DIOS | DENGUE WITHOUT WARNING SIGNS | 21 | 103 | 12 | *NA* | *NA* | 85 | 2 | 314 | 45 | 1 | 12 | 128 | 108 | 174 | 81 | 197 | 104 | 139 | 346 | 1641 | 1010 | 319 | 949 |
| MADRE DE DIOS | DENGUE WITH WARNING SIGNS | *NA* | *NA* | *NA* | *NA* | *NA* | *NA* | *NA* | *NA* | *NA* | 797 | 2 | 8 | 5 | 10 | 5 | 26 | 9 | 11 | 22 | 102 | 25 | 10 | 20 |
| MADRE DE DIOS | SEVERE DENGUE | *NA* | *NA* | *NA* | *NA* | *NA* | *NA* | *NA* | *NA* | *NA* | *NA* | 2938 | 1820 | 1934 | 2088 | 1031 | 743 | 355 | 415 | 866 | 5655 | 2198 | 1004 | 2672 |
| PASCO | DENGUE WITH WARNING SIGNS | *NA* | *NA* | 22 | 1 | 6 | 3 | *NA* | 2 | 30 | 29 | *NA* | 2 | 6 | 14 | 21 | 10 | 14 | 1 | 1 | 36 | 16 | 40 | 80 |
| PASCO | SEVERE DENGUE | *NA* | *NA* | *NA* | *NA* | *NA* | *NA* | *NA* | *NA* | *NA* | *NA* | *NA* | 85 | 74 | 1 | 12 | 1 | 36 | 3 | 2 | *NA* | 10 | 2 | 2 |
| PASCO | DENGUE WITHOUT WARNING SIGNS | *NA* | *NA* | *NA* | *NA* | *NA* | *NA* | *NA* | *NA* | *NA* | *NA* | *NA* | *NA* | *NA* | 41 | *NA* | 21 | *NA* | *NA* | *NA* | *NA* | 351 | 574 | 414 |
| PIURA | DENGUE WITHOUT WARNING SIGNS | 2620 | 167 | 101 | 15 | 37 | 1 | 865 | 282 | 5 | 1 | 28 | 9 | 163 | 337 | 218 | 2629 | 1364 | 6137 | 33 | 4 | 18 | 242 | 1422 |
| PIURA | DENGUE WITH WARNING SIGNS | *NA* | 11546 | *NA* | 1711 | *NA* | 50 | *NA* | *NA* | 1697 | 4029 | 13 | 174 | 3 | 6 | 7 | 51 | 36 | 141 | 1 | 1 | 2 | 10 | 51 |
| PIURA | SEVERE DENGUE | *NA* | *NA* | *NA* | *NA* | *NA* | *NA* | *NA* | *NA* | *NA* | *NA* | 8352 | *NA* | 1015 | 1633 | 2450 | 17361 | 6210 | 37996 | 491 | 65 | 105 | 3820 | 10677 |
| SAN MARTIN | DENGUE WITHOUT WARNING SIGNS | 218 | 179 | 42 | 46 | 6 | 2 | 170 | 677 | 541 | 1 | 5 | 112 | 407 | 325 | 194 | 47 | 74 | 99 | 18 | 504 | 788 | 668 | 921 |
| SAN MARTIN | DENGUE WITH WARNING SIGNS | *NA* | *NA* | *NA* | *NA* | 571 | 170 | *NA* | *NA* | *NA* | 449 | 302 | 2 | 33 | 9 | 9 | 1 | 2 | 361 | 80 | 15 | 32 | 15 | 24 |
| SAN MARTIN | SEVERE DENGUE | *NA* | *NA* | *NA* | *NA* | *NA* | *NA* | *NA* | *NA* | *NA* | *NA* | *NA* | 1323 | 1882 | 874 | 1371 | 172 | 259 | *NA* | *NA* | 1450 | 4135 | 3849 | 3325 |
| TUMBES | DENGUE WITHOUT WARNING SIGNS | 192 | 81 | 13 | 50 | 9 | 2 | 243 | 79 | 51 | 830 | 1 | 2 | 66 | 21 | 500 | 2093 | 49 | 69 | 2 | 10 | 74 | 72 | 36 |
| TUMBES | DENGUE WITH WARNING SIGNS | *NA* | 1722 | *NA* | *NA* | 1543 | 181 | *NA* | *NA* | *NA* | *NA* | 4 | 102 | 1 | 1 | 9 | 3 | 1 | 7 | 62 | 498 | 1 | 3 | 687 |
| TUMBES | SEVERE DENGUE | *NA* | *NA* | *NA* | *NA* | *NA* | *NA* | *NA* | *NA* | *NA* | *NA* | 1172 | *NA* | 525 | 228 | 1191 | 5322 | 1039 | 4069 | *NA* | *NA* | 3018 | 1380 | *NA* |
| UCAYALI | DENGUE WITHOUT WARNING SIGNS | 97 | 682 | 13 | 182 | 14 | 69 | 1 | 5 | 19 | 6 | 1 | 468 | 2739 | 379 | 555 | 198 | 249 | 207 | 69 | 41 | 1372 | 432 | 696 |
| UCAYALI | DENGUE WITH WARNING SIGNS | *NA* | *NA* | 2964 | *NA* | 1399 | *NA* | 173 | 177 | 912 | 1066 | 120 | 16 | 74 | 16 | 15 | 7 | 12 | 18 | 8 | 6 | 50 | 22 | 27 |
| UCAYALI | SEVERE DENGUE | *NA* | *NA* | *NA* | *NA* | *NA* | *NA* | *NA* | *NA* | *NA* | *NA* | *NA* | 1286 | 8243 | 664 | 923 | 145 | 746 | 554 | 240 | 166 | 8359 | 2686 | 4119 |
